# A Flow Cytometry-Based Assessment of the Genomic Size and Ploidy Level of Wild *Musa* Species in India

**DOI:** 10.3390/plants12203605

**Published:** 2023-10-18

**Authors:** Rithesh B. Natarajan, Pooja Pathania, Hardeep Singh, Anuradha Agrawal, Rajkumar Subramani

**Affiliations:** 1 Division of Plant Genetic Resources, ICAR-Indian Agricultural Research Institute, Pusa Campus, New Delhi 110012, India; 2 ICAR-National Bureau of Plant Genetic Resources, Pusa Campus, New Delhi 110012, India; 3 Indian Council of Agricultural Research, Pusa Campus, New Delhi 110012, India

**Keywords:** banana, chromosome, flow cytometry, genome size, *Musa*, nuclear DNA, ploidy

## Abstract

The genome size variation is an important attribute in evolutionary and species characterization. *Musa* L. is regarded as one of the taxonomically complicated genera within the order Zingiberales, with more than 75 species from wild seeded to seedless cultivars that may be diploid, triploid or tetraploid. The knowledge of total nuclear DNA content in terms of genome size and ploidy level in wild species of *Musa* is absolutely important in evolutionary and genomic studies. Methods: In this paper, chromosome spreading was performed via protoplast isolation and a fast air-dry dropping method and flow cytometry were used with *Raphanus sativus* L. (Brassicaceae) as a standard for ploidy and genome size estimation. Results: The results showed that genome size (2C) varied amongst *Musa* species, based on the ratio of G1 peak positions. The lowest genome size (2C) was found in *M. balbisiana* var. *andamanica* (1.051 ± 0.060 pg) and the highest genome size (2C) was recorded for *Musa* ABB.cv. Meitei-hei (1.812 ± 0.108 pg) for the section *Eumusa*. Among the species belonging to the section *Rhodochlamys*, *M. rosae* had the lowest 2C content of 1.194 ± 0.033 pg whereas the highest nuclear DNA content (2C) was observed in *M. velutina* (1.488 ± 0.203 pg). Cytogenetic analysis revealed that the chromosome number of 14 wild *Musa* species was 2n = 22, while 1 species—*Ensete glaucum*—showed a chromosome number of 2n = 18 (diploid), and for 3 species, the chromosome number was 2n = 33 (triploids). An association study based on the Pearson correlation coefficient showed 2C nuclear DNA content was significant and positively correlated with ploidy level (R = 0.9) and chromosome number (R = 0.84). Conclusions: The present study provides reliable information on the genome size and ploidy level of wild *Musa* species from the Indian region through flow cytometric analysis, which could be further utilized in taxonomic and crop improvement programs. For the first time, the nuclear DNA content of eight wild diploid and three triploid Indian species were estimated and reported. Genome size could be an effective indicator in identification of species and evolutionary studies in *Musa* with varying ploidy levels and morphological similarities.

## 1. Introduction

Banana (*Musa* spp.) represents one of the world’s major staple fruit crops and is an important export commodity for millions of people living in tropical and subtropical regions [1]. It is a significant export commodity for various developing nations and plays a crucial role in their national trade, contributing to their socioeconomic development. In 2021, the annual global banana production was reported to be around 124.97 million tons (mt), out of which the highest amount of 33.06 mt was produced by India (https://www.fao.org/faostat/en/#data, accessed on 8 May 2013). Bananas are monocotyledonous plants belonging to the family Musaceae under the order Zingiberales with three genera: *Musa* L., *Ensete* Horan., and *Musella* (Franch.) H.W. Li. [2] The largest genus, *Musa*, comprises about 75 species and is mainly distributed in Southeast Asian region [1,3]. *Ensete*, a smaller genus, is present in both Asia and Africa [4,5], whereas the monotypic genus *Musella* is native to Southwest China [6]. The genus *Musa* was divided into five sections—*Australimusa*, *Callimusa, Eumusa*, *Incertae,* and *Rhodochlamys*—based on their chromosome number and morphological characters [4,7]. This traditional classification of *Musa* has been reappraised based on numerous molecular studies merging *Eumusa* and *Rhodochlamys* into the section *Musa* and *Australimusa* and *Callimusa* into the section *Callimusa* [3,8]. The majority of the edible banana cultivars have originated from inter- and intra-specific hybridization of two wild diploid species (2n = 2x = 22), namely *M. acuminata* Colla (A genome) and *M. balbisiana* Colla (B genome). Other species, such as *M. schizocarpa* N.W. Simmonds (S genome) and *M. textilis* Nee (T genome), also contributed to the origin of some edible banana clones to some extent [9,10]. The combinations of these genomes have resulted in various genotypes of cultivated edible banana clones, exhibiting different genomic composition and ploidy levels such as AA, AB, AAA, AAB, ABB, AAAB, AABB, and ABBB [11]. In India, the genus is represented by 37 wild taxa—of which 20 are endemic—and is largely distributed in northeastern states and followed by Western Ghats, Eastern Ghats, and Andaman and Nicobar Islands [12,13]. However, the family Musaceae is well known for its vast genetic diversity and thought to have originated from south and Southeast Asia [13,14]. Wild seeded bananas play a vital role in enhancing banana crop improvement, serving as a valuable repository of a diverse gene pool that harbors resistant genes against significant diseases, pests, and abiotic stresses. Despite being a major fruit crop, only *M. acuminata* and *M. balbisiana* have been used for breeding novel varieties [15,16]. Recently, a search for traits in evolutionarily related *Musa* species revealed a socioeconomic importance of wild bananas in banana breeding programs. However, to our knowledge, only a few wild species have been studied and to a limited extent, so little is known about their nuclear genomes.

The use of molecular tools provides an efficient method for determination of genome composition and has been extensively applied in taxonomic classification of *Musa* species. One of the basic characteristics of the taxonomic studies of higher plants is their nuclear genome size, which serves as an essential feature in classification and provides useful insights about various species [17]. The genome size or DNA C value is essential and remains a key component in the genomic studies, phylogeny, and species classification [18,19]. Considering genome size in conjunction with chromosome numbers and ploidy status can offer valuable perspectives on the evolutionary relationships between closely related taxa in wild plants [20]. Genome size can have significant variation within the same genus and among different individuals within a species [19,21]. The nuclear genome size (equal to 1C) can be expressed either in picograms (pg) or megabase pairs (1 pg = 978 Mbp), corresponding to the method of estimation [22]. So far, genome size in *Musa* spp. has been estimated to be relatively small (1C, ~ 600 Mbp) and divided between 11 chromosomes [23]. These estimations were focused only on *M. acuminata*, *M. balbisiana*, triploid, and tetraploid cultivars. Consequently, there is an urgent necessity to extend the knowledge of nuclear genome sizes to the other *Musa* species as well. Although different methods have been used to estimate genome sizes in plants, flow cytometry (FC) serves as a primary method for the estimation of nuclear DNA content (2C) and ploidy. In comparison to the traditional chromosome-counting technique, FC holds an advantage as it allows for the screening of numerous plants in a short timeframe [23,24]. It has also become the method of choice due to the ease of sample preparation, relatively low cost, and the ability to analyze high numbers of nuclei; it can also be applied to any plant tissue [25]. FC determines the relative DNA content of each nucleus by quantifying the fluorescence emitted by each stained nucleus; the estimation also requires a species with known genome size as an internal or external standard [24]. Furthermore, the genome size also plays a significant role in distinguishing hidden *Musa* species where the variation in ploidy is known. Therefore, this study was conducted to determine the nuclear DNA content, ploidy level, and chromosome count of wild *Musa* in India with the aim to investigate the genome size variation using FC analysis.

## 2. Results

### 2.1. Chromosome Number Analysis

**Figure 1:**
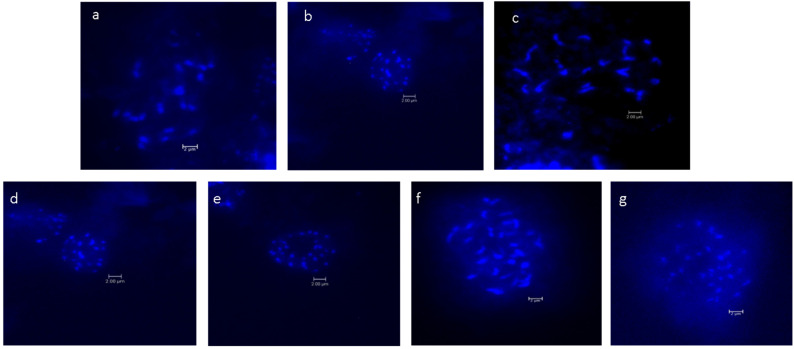
Mitotic metaphase chromosomes of *Musa*: (**a**). *M. balbisiana* var. *andamanica,* (**b**). *M. indandamanensis*, (**c**). *M. sikkimensis*, (**d**). *M. paramjitiana*, (**e**). *M*. AAB var. Champacolla, (**f**). *M*. ABB var. Meitei-hei, (**g**). *M*. AAA var. Vaibalha (scale bar 2 um).

The chromosome numbers of the *Musa* species and cultivars are presented in Table 1. Images of metaphase spread reported for the first time for seven *Musa* species and cultivars are shown in Figure 1. The chromosome count revealed that 14 wild *Musa* species were confirmed to be diploids, comprising the sections *Eumusa* and *Rhodochlamys* with chromosome number 2n = 22. In the section *Eumusa*, 3 *Musa* species were triploids with a chromosome number 2n = 33. In the genus *Ensete*, the species *Ensete glaucum* showed a different chromosome number, 2n = 18.

### 2.2. Ploidy Level Analysis

This study showed that out of 18 accessions, 14 *Musa* accessions were diploids (2n = 2x = 22) and 3 were triploids (2n = 3x = 33). One species of genus *Ensete, Ensete glaucum*, was diploid (2n = 2x = 18) (Table 1). A significant difference was observed in fluorescent intensity corresponding to different ploidy levels; for each value of ploidy, the fluorescent intensity of the G1 peak differed significantly between diploids and triploids. However, the study also showed that most of the wild *Musa* accessions in both sections (*Eumusa* and *Rhodochlamys*) were diploid, but three cultivars in the section *Eumusa* were triploids.

### 2.3. Variation in Genome Size Values

**Figure 2:**
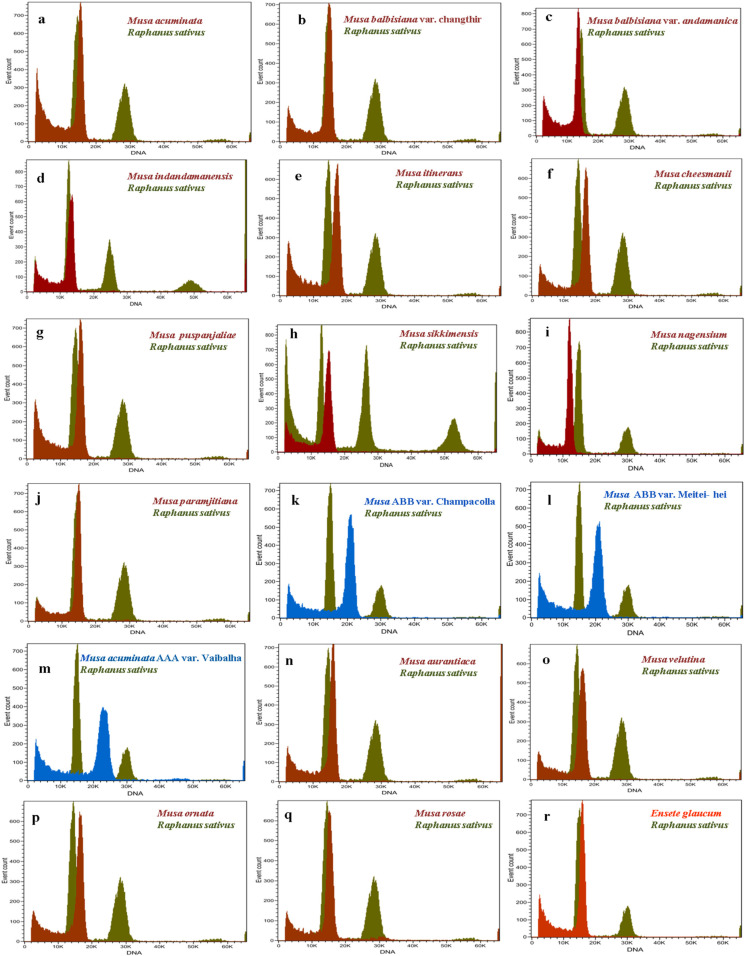
Histogram of relative nuclear DNA content, with the dominant peak corresponding to G1 nuclei of *Musa* and *Raphanus sativus* obtained after flow cytometry analysis. (**a**). *M. acuminata*, (**b**). *M. balbisiana* var. Changthir, (**c**). *M. balbisiana* var. *andamanica*, (**d**). *M. indandamanensis*, (**e**). *M. itinerans*, (**f**). *M. cheesmanii*, (**g**). *M. puspanjaliae*, (**h**). *M. sikkimensis*, (**i**). *M. nagensium*, (**j**). *M. paramjitiana*, (**k**). *M*. AAB cv. Champacolla, (**l**). *M.* ABB cv. Meitei-hei, (**m**). *M.* AAA var. Vaibalha, (**n**). *M.aurantiaca*, (**o**). *M.velutina*, (**p**). *M.ornata*, (**q**). *M. rosae*, and (**r**). *Ensete glaucum*.

The amount of nuclear DNA was estimated after flow cytometric analysis of propidium iodide-stained nuclei, and the means 2C values corresponding to *Musa* species are presented in Table 1. The histograms of relative nuclear DNA content with two dominant peaks corresponding to G1 nuclei of *Musa* sp. and *Raphanus sativus* are shown in Figure 2. The coefficient of variation (CV) for G1 of all *Musa* sample peaks was less than five, indicating the good quality of sample preparation and reliability of the results. The analysis revealed the nuclear DNA content as 2C values based on the ratio of G1 peak positions, which ranged from 1.051 to 1.812 pg for the section *Eumusa*. The lowest nuclear DNA size was observed in the diploid taxon *M. balbisiana* var. *andamanica* with 1.051 pg and the highest nuclear DNA content was recorded for the triploid *Musa* ABB var. Meitei-hei with 1.812 pg in the *Eumusa* section. The section *Rhodochlamys* showed a similar range of nuclear DNA content, ranging from 1.194 to 1.488 pg. The lowest nuclear DNA content was recorded for *M. rosae* (1.194 pg) and the highest for *M. velutina* (1.488 pg). *Ensete glaucum*, an outgroup in the section, had 2C nuclear DNA content of 1.349 pg. The FC analysis of 2C nuclear DNA contents of two sections, *Eumusa* and *Rhodochlamys*, showed overlapping results with little difference. The three triploid *Musa* accessions—namely, *Musa* AAB. var. Champacolla, *Musa* ABB. var. Meitei-hei, and *Musa acuminata* AAA. Vaibalhla—showed a higher range of nuclear DNA content of 1.802, 1.812, and 1.741 pg, respectively, than diploid species from both sections. The mean 2C values of *Raphanus sativus* and wild *Musa* species from the *Eumusa* and *Rhodochlamys* sections were close together, and a merged inflorescence peak was observed when *Raphanus sativus* was used as a reference standard. Considerable variation in nuclear DNA content was also observed among *Musa* accessions representing both sections.

**Figure 3:**
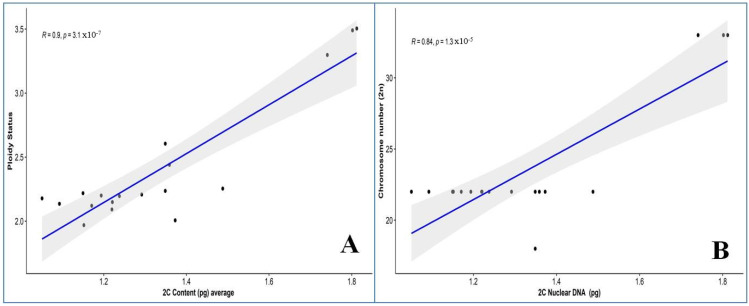
Relationship between nuclear 2C DNA content of the *Musa* species (**A**) with ploidy status, (**B**) with chromosome number.

The study showed the calculated average genome size (1C content) of all *Musa* accession, which ranged from 514.1 to 885.9 Mbp. The *Musa balbisiana* var. *andamanica* showed the lowest 1C content of 514.1 Mbp among all the accessions, while the *Musa* ABB. var. Meitei-hei showed the highest 1C content with 885.9 Mbp. Based on the Pearson correlation coefficient, the 2C nuclear DNA content showed a strong significance and positively correlated with ploidy level (R = 0.9) (Figure 3A) and chromosome number (R = 0.84) (Figure 3B) among the *Musa* species.

## 3. Discussion

Southeast Asia is considered one of the world’s biodiversity hotspots for *Musa* spp. India has a significant amount of banana genetic diversity in the wild and is known as the center of origin for interspecific hybrids worldwide. The basic genomic information of wild *Musa* species originated from India would be of much importance for future banana improvement programs. We have determined the chromosome number, ploidy level, and genome size of wild *Musa* species in India.

### 3.1. Chromosome Number and Ploidy Status

The number of somatic chromosomes of *Musa* will be important when designating and confirming the species ploidy level. The hard and rigid cell walls from the root system of cultured *Musa* species were a limiting factor for preparing chromosome spread [26] through the traditional squash method, so protoplast isolation and fast air-dry dropping chromosome preparation was performed in this study. Cytogenetic analysis confirmed the chromosome number of wild *Musa* species from sections *Eumusa* and *Rhodochlamys* as diploids (2n = 2x = 22), and triploid cultivars were found to be 2n = 3x = 33, ensuring the use of basic chromosome numbers for classifying the *Musa* species [4]. The genus *Ensete* and its species with monocarpic habits are sister genera close to *Musa* in the family Musaceae, but with the basic chromosome number x = 9 [4,27]. The *Ensete glaucum* is a diploid with a chromosome number of 2n = 2x = 18. Although chromosome counting is accurate and applied primarily to plants with large chromosomes, challenges remain with regard to the banana’s chromosome; due to its small, indistinguishable size and high level of metaphase condensation, it is hard to obtain a high quality of chromosome spread [27,28]. There is limited information concerning the structural characteristics of chromosomes and karyotypes for the *Musa* species, and detailed information about the chromosomes’ number and their size, shape, and arrangement within this genus remains scarce [29]. A variety of indirect methods for determining banana ploidy level have been reported [30,31]. However, this method depends on statistical analysis and is not accurate. In the present study, the flow cytometry method is further applied to confirm the ploidy status of *Musa* spp.

FC provides a rapid and efficient method for large-scale determination of ploidy level in Musaceae [32]. Christelová et al. [33] used the flow cytometry method to rapidly estimate and genotype nearly half of the ITC accessions (495 accessions) of the global *Musa* germplasm collection. In the present study, the ploidy level was determined by measuring the peak position of sample nuclei in comparison with the peak position of reference nuclei, supplemented with DAPI during flow cytometry analysis. The fluorescence intensity was significantly different for each *Musa* species, in which the ploidy level increased with increased fluorescent intensity. We also observed a shift in the peak position to the right as an increase in the ploidy level of *Musa* species. Furthermore, the flow cytometry analysis for determining ploidy reconfirms via the chromosome counting method that the ploidy status of the 14 *Musa* and one *Ensete* species are diploids and three cultivars are triploids. Therefore, the knowledge on *Musa* ploidy is valuable for taxonomy and crop improvement as it plays an important role in classifying individual species.

### 3.2. Analysis of Genome Size

Nuclear genome size is considered a basic characteristic in classifying the species and providing valuable information on taxonomy and the evolutionary relationship of the higher plant [16,34]. Earlier reports on the flow cytometry technique also suggested that it could be applied to the analysis of the nuclear DNA content of unknown samples with respect to known reference standards and their ability to differentiate between closely related species, even of various ploidy levels [16,23]. The present results showed that the nuclear DNA content of *Musa balbisiana* (1.046 pg) was smaller than *Musa acuminata* (1.220 pg). Kamaté et al. [35] reported 1.16 pg for *Musa balbisiana* (BB genome), 1.27 pg for *Musa acuminata* (AA genome), and also recorded the difference at their subspecies level of approximately 11%. Our present study used genome size as a key component to determine the intra- and interspecific variation and the relationship between wild *Musa* species in India. Current analysis showed a wide range of nuclear DNA content in the sections *Eumusa* and *Rhodochlamys*. Several researchers noted a close relationship between species of the sections *Eumusa* and *Rhodochlamys*, both morphologically [27] and at molecular level [36]. Our results also showed a close relationship between two investigated sections. The high range of variation in the nuclear DNA content (2C/pg) can also enrich the *Musa* C- value database. Čížková et al. [36] studied the nuclear DNA content of 21 diploid accessions representing sections of the genus *Musa* and showed the 2C DNA content in the section *Musa* ranged from 1.217 to 1.315 pg. Investigations on the genome size of genera *Musa* and *Ensete* showed overlap among their genome sizes, with a range from 0.61 to 0.69 pg/1C in *Eumusa* and from 0.6 to 0.66 pg/1C in *Rhodochlamys* [16,17]. Similarly, our present study showed 2C nuclear DNA values in the *Eumusa* and *Rhodochlamys* sections exhibited a degree of overlap with little difference. The three triploid *Musa* accessions showed a higher range of nuclear DNA content than diploid species in both sections. Our study also showed the calculated average 1C value of all *Musa* species ranged from 514.1 Mbp to 885.9 Mbp in both the *Eumusa* and *Rhodochlamys* sections. Lysảk et al. [37] also observed variation in the genome size of the genus *Musa*. The FC method previously used to estimate the genome size resulted in a clear distinction between *M. acuminata* and *M. balbisiana* with genome size of ~0.6–0.64 pg/1C and ~0.55–0.57 pg/1C, respectively, and is closely related to our findings.

The positive correlation between chromosome number and genome size in the Indian wild *Musa* species is similar to that of other studies on cultivated and wild species of *Musa* [24]. The intraspecific variation in genome size is attributed to variation in the tandem repeats, transposable elements (TEs), polyploidization, and recombination rate [38,39]. Likewise, in *Musa* 55% of the genome is composed of various repetitive and non-coding sequences [40], while the nuclear genome in bananas contains coding genes every 6.4 to 6.9 kb [41,42]. The variation in repetitive sequence was found to be a major factor in differentiation in the genus *Musa*, even at subspecies level [43,44]. The difference in genome size between *M. acuminata* and *M. balbisiana* has been attributed to a variation in the copy number of repetitive DNA sequences such as “Monkey” [45] and Radka5 [46]. The systematic studies on the newly described endemic species of *Musa* from India revealed all of them belong to either the *balbisiana* or *acuminata* complex. Along with sequence data, the nuclear genome size of newly described *Musa* species will provide valuable information on genomic studies, evolutionary relationship, and species classification.

## 4. Materials and Methods

### 4.1. Plant Material

In vitro-rooted plantlets of 17 *Musa* and one *Ensete* species were obtained from Tissue Culture and Cryopreservation Unit, ICAR-National Bureau of Plant Genetic Resources (NBPGR), New Delhi (Table 2). *Raphanus sativus* cv Saxa (2C = 1.11 pg) reference standard was obtained from Institute of Experimental Botany, Czech Republic.

### 4.2. Cytogenetic Studies

Mitotic metaphase spreads were prepared according to the method described by Doleželová et al. [47], with minor modifications. Actively growing *Musa* root tips were pre-treated in 0.002 M of 8-hydroxyquinoline for 4 h at room temperature and then fixed for 24 h in 3:1 ethanol: acetic acid overnight and stored in 70% ethanol. Fixed roots were washed with 1X enzyme buffer solution (40 mL of 100 mM Citric Acid, 60 mL of 100 mM Trisodium Citrate and pH adjusted to 4.8, and diluted 1:10 in distilled water) and digested in the enzyme solution (containing 2% *w*/*v* cellulase, 3% *v*/*v* pectinase and 1X enzyme buffer) and kept in a bath-marie at 37° C for 2.5 h. Digested root tips were washed thrice with 1X enzyme buffer and cell suspension was prepared using 3:1 acetic acid: ethanol. The suspension was dropped onto a clean dry ice-cold microscopic slide placed on a hot plate (55 °C) from a height of 30 to 40 cm. The suspension was allowed to spread out and air dry [48]. Finally, the slides were examined under a fluorescence microscope (Leica DM6 B) and the images captured using the Leica application suite (LAS) X 3.8 software.

### 4.3. Flow Cytometry—Determination of Nuclear Genome Size and Ploidy Status

A small amount of leaf sample (~50 mg) was taken in a glass Petri dish and 500 μL of extraction buffer (CyStain PI absolute P kit, Sysmex, Germany) was added. The sample was finely chopped using a sharp scalpel blade for 30 s. and filtered through 20 μm CellTrics filter into a sample tube. For genome size estimation, the chopped samples were incubated in 2 mL of staining solution, supplemented with Propidium Iodide and Rnase (CyStain PI Absolute P kit, Sysmex, Germany), and incubated in the dark on ice for 30 min. The sample was analyzed in a flow cytometer (CyFlow Ploidy Analyser, Sysmex, Germany) under red channel (532 nm). *Raphanus sativus* was used as a reference standard. The nuclear genome size of samples was determined using the formula given by Doležel et al. [49]. 2C nuclear DNA content = Reference 2C value × Sample 2C mean peak position Reference 2C mean peak position.

The mean nuclear DNA content was then calculated for each plant. The genome size, which represents one copy of nuclear genetic information (equal to 1C), was further determined considering 1 pg = 0.978 × 10^9^ bp [22].

1C (Mbp) = nuclear DNA content (pg) × (0.978 × 10^9^)/2

To improve accuracy, the nuclear DNA content was determined for each *Musa* sample in three replicates, enabling the standard error to be calculated.

For ploidy determination, staining buffer supplemented with DAPI was added to the sample after the nuclei extraction step. The experiment was performed in three replicates. Relative fluorescence intensity of stained nuclei was analyzed using a flow cytometer (CyFlow Ploidy Analyser) under a UV channel. Standardization of flow cytometry was carried out using a confirmed diploid *Musa* species, *Musa balbisiana* (ITC-0565), obtained from the *Musa* Germplasm Transit Centre. *Musa balbisiana* was used as reference standard for ploidy screening to assess the ploidy status of other species under analysis. The ploidy of *Musa* species was determined by comparing the fluorescence values between the peak positions of samples to that of reference standards. The *Musa* samples were analyzed in the flow cytometry under UV channel and a minimum of 5000 particles were measured for both reference and samples.

Analyses of variance (ANOVA) were performed to analyze the variation in nuclear DNA size (R 4.2.1 software- Stat package and agricolae). The significance level = 0.001 was used. Pearson correlation analysis was performed to learn the associations among nuclear DNA content, chromosome number and ploidy status using R 4.2.1 software (Package-ggpubr and ggplot2).

## 5. Conclusions

Our study showed that the identification of genome size and ploidy level is important for taxonomy and crop improvement programs. There was no clear gap in nuclear DNA content between the sections *Eunusa* and *Rhodochlamys*. Furthermore, this study showed that nuclear DNA content in relation with the chromosome numbers and ploidy status provided valuable information on the evolutionary relationships and characterization of *Musa* species. We observed a significant difference in the nuclear genome size and ploidy status of wild *Musa* species belonging to the *Eumusa* and *Rhodochlamys* sections. Our study also showed the positive correlation between nuclear DNA content and ploidy level and emphasizes that flow cytometry can be used for rapid and precise estimation of ploidy as a metric for genome characterization.

## Figures and Tables

**Table 1:** Nuclear DNA content, genome size, ploidy level, and chromosome count of investigated accessions from *Musa* and *Ensete* genera and two standard species (*Raphanus sativus* and *Musa balbisiana* cultivar ITC0565).

Section	Species	2C Nuclear DNA (pg) Average ± SE	Genome Size 1C (Mbp)	Ploidy	Chromosome Number (2n)
	*Raphanus sativus*	1.11 ± 0.00	542.8	2	18
*Eumusa*	*Musa acuminata*	1.220 ± 0.087	596.6	2	22
	*Musa balbisiana* (ITC0565)	1.046 ± 0.034	511.4	2	22
	*Musa balbisiana* var. Changthir	1.150 ± 0.071	562.5	2	22
	*Musa balbisiana* var. *andamanica*	1.051 ± 0.060	514.1	2	22
	*Musa indandamanensis*	1.171 ± 0.080	572.7	2	22
	*Musa itinerans*	1.349 ± 0.031	659.7	2	22
	*Musa cheesmani*	1.373 ± 0.232	671.5	2	22
	*Musa puspanjaliae*	1.221 ± 0.046	596.9	2	22
	*Musa sikkimensis*	1.359 ± 0.072	664.6	2	22
	*Musa nagensium*	1.093 ± 0.016	534.3	2	22
	*Musa paramjitiana*	1.152 ± 0.045	563.3	2	22
	*Musa* AAB var. Champacolla	1.802 ± 0.063	881.2	3	33
	*Musa* ABB var. Meitei-hei	1.812 ± 0.108	885.9	3	33
	*Musa acuminata* AAA var. Vaibalhla	1.741 ± 0.109	851.3	3	33
*Rhodochlamys*	*Musa aurantiaca*	1.238 ± 0.029	605.2	2	22
	*Musa velutina*	1.488 ± 0.203	727.7	2	22
	*Musa ornata*	1.292 ± 0.027	631.6	2	22
	*Musa rosae*	1.194 ± 0.033	584.0	2	22
	*Ensete glaucum*	1.349 ± 0.093	659.8	2	18

Statistical analysis was performed using mean values calculated for individual plants (n = 3) and significance level α = 0.01.

**Table 2:** List of *Musa* and *Ensete* species used in the present study.

S.No	Species	Accession Detail	Genomic Constitution	Section	Place of Collection	Crop/Special Characteristics
1	*Musa acuminata*	IC633381	AA	Eumusa	Mizoram	Seeded Banana
2	*Musa balbisiana*	ITC0565	BB	Eumusa	Belgium	Ploidy reference standard
3	*Musa balbisiana* var. changthir	IC833382	BB	Eumusa	Mizoram	Seeded Banana
4	*Musa balbisiana* var. *andamanica*	IC630992	BB	Eumusa	Andaman and Nicobar Islands	Seeded Banana
5	*Musa indandamanensis*	IC631162	-	Eumusa	Andaman and Nicobar Islands	Wild Banana with 11 mts height, sweet with orange fruit plup
6	*Musa itinerans*	-	-	Eumusa	-	Wild banana with Pink Fruit and spreading rhizomatous roots
7	*Musa cheesmani*	-	-	Eumusa	-	Wild banana with Black hard seed
8	*Musa puspanjaliae*	-	-	Eumusa	-	Largest seeded banana
9	*Musa sikkimensis*	-	-	Eumusa	Sikkim	Red striped leaves
10	*Musa nagensium*	IC627969	-	Eumusa	Arunachal Pradesh	Wild Banana
11	*Musa paramjitiana*	IC628650	-	Eumusa	Andaman and Nicobar Islands	Sweet and sour tasting fruit with boat shape and bulb shaped seeds
12	*Musa* AAB. var. Champacolla	-	AAB	Eumusa	-	Cultivar
13	*Musa* ABB. Meitei-hei	-	ABB	Eumusa	-	Cultivar
14	*Musa acuminata* AAA. Vaibalhla	-	AAA	Eumusa	-	Cultivar
15	*Musa aurantiaca*	IC627978	-	Rhodochlamys	Nagaland	Dwarf Banana with bright orange inflorescence
16	*Musa velutina*	IC636539	-	Rhodochlamys	Assam	Pink Fruiting Banana/Hairy Banana
17	*Musa ornata*	IC633379	-	Rhodochlamys	Mizoram	Flowering Banana
18	*Musa rosae*	-	-	Rhodochlamys	-	Wild ornamental Banana
19	*Ensete glaucum*	IC633380	-		Mizoram	Snow Banana

## Data Availability

All the data are available in the manuscript.
